# Combined Effect of *TLR2* Gene Polymorphism and Early Life Stress on the Age at Onset of Bipolar Disorders

**DOI:** 10.1371/journal.pone.0119702

**Published:** 2015-03-19

**Authors:** José Oliveira, Bruno Etain, Mohamed Lajnef, Nora Hamdani, Meriem Bennabi, Djaouida Bengoufa, Aparna Sundaresh, Arij Ben Chaabane, Frank Bellivier, Chantal Henry, Jean-Pierre Kahn, Dominique Charron, Rajagopal Krishnamoorthy, Marion Leboyer, Ryad Tamouza

**Affiliations:** 1 INSERM, U1160, Hôpital Saint Louis, Paris, France; 2 INSERM, U955, Psychiatrie Génétique, Créteil, France; 3 Laboratoire Jean Dausset and LabEx Transplantex, Hôpital Saint Louis, Paris, France; 4 Université Paris-Est, Faculté de Médecine, Créteil, France; 5 AP-HP, Pôle de Psychiatrie, DHU PePSY, Hôpitaux Universitaires Henri Mondor, Créteil, France; 6 Fondation FondaMental, Créteil, France; 7 Service de Psychiatrie et Psychologie Clinique, CHU de Nancy, Hôpitaux de Brabois, Vandoeuvre Les Nancy, France; 8 Université Paris Diderot, Sorbonne Paris-Cité, Paris, France; Rutgers University, UNITED STATES

## Abstract

Gene-environment interactions may play an important role in modulating the impact of early-life stressful events on the clinical course of bipolar disorder (BD), particularly associated to early age at onset. Immune dysfunction is thought to be an important mechanism linking childhood trauma with early-onset BD, thus the genetic diversity of immune-related loci may account for an important part of the interindividual susceptibility to this severe subform. Here we investigated the potential interaction between genetic variants of Toll-like receptors 2 (*TLR2*) and 4 (*TLR4*), major innate immune response molecules to pathogens, and the childhood trauma questionnaire (CTQ) in age at onset of BD. We recruited 531 BD patients (type I and II or not otherwise specified), genotyped for the *TLR2 rs*4696480 and *rs*3804099 and *TLR4 rs*1927914 and *rs*11536891 single-nucleotide polymorphisms and recorded for history of childhood trauma using the CTQ. *TLR2* and *TLR4* risk genotype carrier state and history of childhood emotional, physical and sexual abuses were evaluated in relation to age at onset as defined by the age at first manic or depressive episode. We observed a combined effect of *TLR2 rs*3804099 TT genotype and reported sexual abuse on determining an earlier age at onset of BD by means of a Kaplan-Meier survival curve (*p =* 0.002; corrected *p* = 0.02). Regression analysis, however, was non-significant for the *TLR2*-CTQ sexual abuse interaction term. The negative effects of childhood sexual abuse on age at onset of BD may be amplified in *TLR2 rs*3804099 risk genotype carriers through immune-mediated pathways. Clinical characteristics of illness severity, immune phenotypes and history of early life infectious insults should be included in future studies involving large patient cohorts.

## Introduction

Classically viewed as a cyclical disease, bipolar disorder (BD) is now seen as a multi-systemic, progressive chronic illness whose clinical manifestations can be underpinned by biological and environmental factors interacting in a complex manner [1,2]. This severe disorder is associated with a significant burden of psychiatric and other medical comorbidities as well as premature mortality and is the fourth cause of disease incapacity worldwide [3,4]. In addition, BD is a highly heterogeneous disorder for which identification of underlying mechanisms as well as definition of personalized therapeutic interventions is still elusive. Efforts to define homogenous subgroups have in particular enabled the identification of early-onset BD as being more familial as well as clinically and biologically more homogenous than late-onset subforms [5,6]. Admixture analyses performed in different population groups have consistently identified early-onset BD as a distinct subset associated with worse clinical outcome; severe symptoms, resistance to treatment, higher rate of suicide attempts and of comorbidities, thus suggesting that early age at onset (AAO) is a proxy for severity of BD [5,7]. In terms of mechanisms, it is noteworthy that early-life psychosocial stressors, notably emotional and sexual abuses are associated to early AAO [8,9]. Such stressful events are also known to induce acute and chronic immune/inflammatory alterations [10–13] possibly leading to an increased vulnerability to diabetes, obesity, cardiovascular disorders, autoimmunity, cancer and neurodegeneration commonly observed in adults with history of childhood maltreatment [14–18]. Of interest is that elevated C-reactive-protein (CRP), as well as high prevalence of cardiovascular disorders, type 2 diabetes mellitus, hypertension and obesity, are observed in early onset BD [19,20] and that some of these conditions even precede the diagnosis of BD in pediatric patients [20]. These epidemiological observations may reflect the immune/inflammatory dysfunctions possibly particularly important in early-onset BD. This could be exemplified by the pro-inflammatory gene expression signature observed in monocytes of 85% of adolescent offspring of BD patients who later developed a mood disorder as compared to the only 19% observed among adolescent healthy controls [21]. One of the proposed mechanisms is that acute stressor-mediated events may induce chronic alterations in immune/inflammatory processes in genetically predisposed individuals [12,13]. Exploring the control of innate immune responses in BD, we recently described associations between genetic variants of Toll-like receptor 2 (*TLR2*) and *TLR4* loci and early-onset BD [22,23].

TLRs are proteins belonging to the pattern recognition receptors’ (PRR) family that initiate inflammation by sensing danger signals derived either from invading pathogens or from endogenous damaged tissue. Among eleven different TLRs identified in humans, the trans-membrane TLR2 and TLR4, the most studied ones, are the first line of immune defense against bacteria, viruses, fungi and parasites [24]. Expressed both in the periphery and in the central nervous system (CNS) as well as in the maternal-fetal interface, they are able to initiate immediate immune responses against invading pathogens with potential implications in neuropathological processes [25–27]. This is of particular relevance since a variety of infectious agents *viz* Borna virus, *Toxoplasma gondii*, influenza or herpes simplex I have been associated with BD [28–31]. The currently assumed mechanism, common to these infectious insults in causing such risk, is defective central/systemic immune/inflammatory responses that interfere with expression of pro-inflammatory cytokines by microglia, the resident immune cells in the CNS known to express the full repertoire of TLRs [32]. Accordingly, strong inflammatory stimulation through the TLR-mediated pathway during gestation in mice is currently regarded as a developmental paradigm of psychiatric disorders [32,33]. These models suggest an immune-mediated two-hit mechanism in which early-life infectious events interacting with a background of genetic susceptibility may install a fragile neuro-immunological homeostasis that is potentially overwhelmed by subsequent traumatic life experiences [33–35].

Based on these observations, we hypothesized that the genetic potential of innate immune/inflammatory responses may variably modulate the impact of psychosocial stressors on the clinical expression of BD, assessed here by AAO used as a proxy of severity. In this cross-sectional study, we investigated potential interactions on AAO of BD between functionally-relevant *TLR2*/*TLR4* genetic variations and reported childhood trauma, both previously showed to be independently associated with early-onset BD.

## Materials and Methods

### Subjects

BD patients (*n* = 531) meeting DSM-IV criteria [36] for BD (type I or II and NOS), admitted to three French university-affiliated psychiatric departments (Paris-Créteil, Bordeaux and Nancy), were interviewed by trained psychiatrists with the French version of the Diagnostic Interview for Genetic Studies (DIGS version 3.0) [37]. All patients were of French descent with at least three grandparents from the mainland of France and were consecutively recruited between February 2006 and January 2010. All patients were euthymic at inclusion defined by having a Montgomery-Åsberg Depression Rating Scale score [38] and a Mania Rating Scale score [39] no more than five. The AAO was defined by the age at which the first mood episode (depressive, manic or hypomanic) occurred and determined by reviewing the medical case notes and retrospective information obtained with the DIGS. The cohort was further divided in two subgroups namely early-onset and late-onset, using the AAO of 22 years as the threshold, determined on the basis of previous admixture analyses of independent sample sets [5]. Written informed consent was obtained from all participating subjects and the institutional ethical committee approved the research protocol. These patients were sampled from one large ongoing study that explored genetic and environmental risk factors of BD. Data regarding the association between *TLR2*/*TLR4* genetic variations and BD have been previously published [22,23] and this was the same for the data regarding the association between AAO and childhood trauma [8]. To be included in this analysis, all cases had to have both a retrospective assessment of the AAO of BD, defined by the age of the first manic or depressive episode, as well as available genotypes for *TLR2* or *TLR4* genetic variations.

### Childhood trauma assessment

History of childhood trauma was obtained using the French version of the Childhood Trauma Questionnaire (CTQ) [40] and was available for 62% of the included patients. The CTQ is a retrospective self-report instrument that examines the traumatic childhood experiences of adults and adolescents. It consists of 28-items, each one rated from 1 (never) to 5 (very often), that measure five types of childhood trauma: emotional abuse, emotional neglect, physical abuse, physical neglect and sexual abuse. A total score from 5 to 25 for each type of abuse is determined allowing the classification in four levels of maltreatment (absence, low, moderate and severe) based on cut-off scores determined for each type of trauma [41]. The CTQ has shown excellent test-retest reliability and convergent validity and the different cut-offs have been shown to have good specificity and sensitivity [41,42].

### *TLR2* and *TLR4* genetic polymorphism studies

Four functionally-relevant *TLR2* and *TLR4* single-nucleotide polymorphisms (SNPs) (*rs*4696480, *rs*3804099 and *rs*1927914, *rs*11536891 respectively) were selected based on previous genetic association studies of BD [22,23]. Genomic DNA was extracted from EDTA-treated peripheral blood samples or B-lymphoblastoid cell lines using the Nucleon BACC3 kit (GE HealthCare, Chalfont St Giles, UK). The four SNPs (*TLR2* intron 1 *rs*4696480 A/T, *TLR2* exon 3 *rs*3804099 C/T and *TLR4* promoter *rs*1927914 A/G and 3’UTR *rs*11536891 C/T) were analyzed using pre-developed TaqMan 5’-nuclease assay kits (Applied Biosystems, Foster City, CA, USA) with allele-specific fluorogenic oligonucleotide probes (C__27994607_10, C__22274563_10, C__2704048_10 and C__31784036_10, respectively), as previously reported.

### Statistical analysis

Gaussian distribution of AAO was tested using the Shapiro-Wilk’s (W = 0.896, *p*<0.001). Non-parametric analyses were carried out, using classical Mann-Whitney *U* test to explore associations between *TLR2* and *TLR4* genetic variants and AAO as a continuous variable, for the purposes of the present study.

Association between AAO and CTQ (physical abuse, emotional abuse and sexual abuse sub-scores) was tested with the Kruskal-Wallis’ one-way analysis of variance using each CTQ sub-score as a discrete variable categorized into absent, low, moderate and severe abuse. A logistic regression analysis was performed to statistically test for the gene-environment interaction i.e. between risk genotype of *TLR2 rs*3804099 and the sexual abuse sub-score of the CTQ on the pattern of AAO of BD (dichotomized into early- and late-onset as described above). Finally, a Kaplan-Meier survival curve was used to analyze the combined effect of two risk variables namely *TLR2 rs*3804099 and CTQ sexual abuse sub-score, on AAO. The statistical power was estimated using the Freedman method for two-sample comparison of survival functions Log-rank test. The estimated power was of 80.7%, 80.5% and 82.3% respectively for the comparison of individuals having the two risk factors (*TLR2 rs*3804099 TT genotype and reported childhood sexual abuse) with the other groups i.e. (i) risk genotype with absence of childhood sexual abuse, (ii) non-risk genotype with reported childhood sexual abuse and (iii) absence of both risk factors. Results were corrected for multiple comparisons by means of the Bonferroni correction using a *p*-value of 0.005 as a marker of significance. All analyses were performed using Stata 12 and SPSS statistics 20.

## Results

Both demographic and clinical characteristics of the study subjects are described in Table 1. 47.5% of BD patients (*n* = 252) had an early AAO (before the age of 22) while 52.5% (*n* = 279) a late AAO. A significant proportion of the patients that completed the CTQ report have suffered from one or other form of childhood abuse (physical, emotional or sexual) of at least low severity (61.7%). Among them, 17.9% reported physical abuse, 48.9% emotional abuse and 30.6% sexual abuse.

**Table 1:** Clinical and demographic characteristics of the 531 bipolar disorder patients.

		Mean (range)	SD
Age at inclusion		42.4 (14–80)	13.1
Age at onset		24.8 (6–67)	9.8
		*n*	%
Sex		531	
	Male	220	41.4
	Female	311	58.6
Diagnosis		531	
	BD I	391	73.6
	BD II	113	21.3
	BD NOS	27	5.1
Age at onset		531	
	Early-onset (< 22 years)	252	47.5
	Late-onset (≥ 22 years)	279	52.5
Physical abuse*		329	
	Absent (5–7)	270	82.1
	Low (8–9)	28	8.5
	Moderate (10–12)	14	4.3
	Severe (13–25)	17	5.2
Emotional abuse*		329	
	Absent (5–8)	168	51.1
	Low (9–12)	74	22.5
	Moderate (13–15)	37	11.2
	Severe (16–25)	50	15.2
Sexual abuse*		327**	
	Absent (5)	227	69.4
	Low (6–7)	40	12.2
	Moderate (8–12)	33	10.1
	Severe (13–25)	27	8.3

* Data not available for the entire cohort (data available for 62% of patients).

** Childhood trauma questionnaire sexual abuse sub-score is missing for two patients.

Genotype and allele frequencies of the studied *TLR2* and *TLR4* genetic variants (Table 2) were in Hardy-Weinberg equilibrium and comparable to those reported in public database for Caucasians (http://www.ncbi.nlm.nih.gov).

**Table 2:** Genotype frequencies of the studied *TLR2* and *TLR4* polymorphisms for the 531 bipolar disorder patients.

		*n*	%
*TLR2 rs*4696480*	TT	141	26.9
	AT	247	47.0
	AA	137	26.1
*TLR2 rs*3804099*	TT	172	32.8
	CT	244	46.5
	CC	109	20.8
*TLR4 rs*1927914**	AA	253	48.1
	AG	210	39.9
	GG	63	12.0
*TLR4 rs*11536891**	TT	375	71.3
	CT	135	25.7
	CC	16	3.0

TLR: Toll-like receptor

* Genotyping missing for six individuals.

** Genotyping missing for five individuals.

### Effects of *TLR2* and *TLR4* genetic polymorphism and CTQ abuse scores on age at onset of bipolar disorder

We found that the *TLR2 rs*3804099 TT genotype was marginally associated with an earlier AAO [*p* = 0.01; corrected *p* (*pc*) = 0.1] with mean AAO of 23.5±9.56 years for patients bearing the TT genotype and 25.5±9.93 years for those carrying the others (CT and CC). No association was noted for the other analyzed polymorphisms (*TLR2 rs*4696480, *TLR4 rs*1927914 and *TLR4 rs*11536891).

Furthermore, we found an association between severity of sexual abuse (patients classified into absent, low, moderate and severe trauma scale categories) and AAO of BD (*p* = 0.002; *pc* = 0.02). The mean AAO for patients reporting absent, low, moderate and severe sexual abuse was respectively 25.5±10.45, 25.3±8.78, 19.8±6.52 and 21.5±6.73. No such association with AAO was noted for both physical and emotional abuse.

### Effect of genotype-environment interaction: effect of *TLR2 rs*3804099 / CTQ sexual abuse score on age at onset of BD

**Fig 1:**
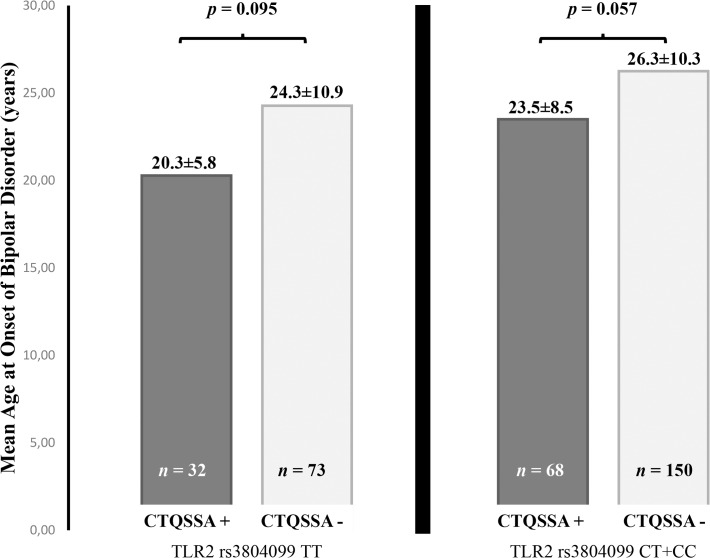
Representation of age at onset of bipolar disorder according to *TLR2* genotype and reported childhood sexual abuse. Mean ± standard deviation of bipolar disorder age at onset is represented according to Toll-like receptor 2 (*TLR2*) *rs*3804099 risk genotype carrier state and reported sexual abuse as defined by the Childhood Trauma Questionnaire (CTQSSA).

**Fig 2:**
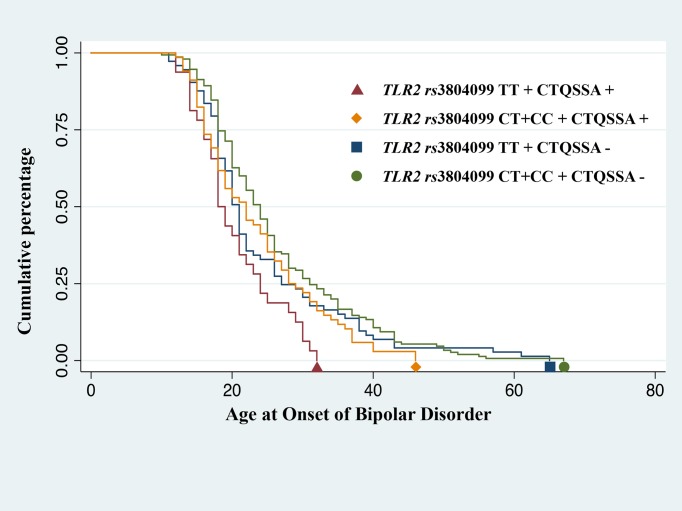
Kaplan-Meier survival curve of age at onset of bipolar disorder. Patients were stratified in four groups according to *TLR2* (Toll-like receptor 2) *rs*3804099 TT risk genotype carrier state and reported childhood sexual abuse as defined by the Childhood Trauma Questionnaire (CTQSSA) and age at onset defined as a time-to-event variable in a Kaplan-Meier survival curve (*p* = 0.002; corrected *p* = 0.02).

Schematic representation of *TLR2 rs*3804099 TT genotype distribution pattern and sexual abuse sub-score as a function of AAO is shown in Fig. 1. No relationship between *TLR2 rs*3804099 polymorphism and childhood sexual abuse on AAO of BD was observed in the logistic regression analysis. Introducing gender as a covariate in the model did not modify the observed results (data not shown) (see below in discussion section). However, using AAO of BD as a “time to event” variable in a Kaplan-Meier survival curve, we found that the presence of both *TLR2 rs*3804099 TT risk genotype and low to severe sexual trauma had a cumulative effect on AAO of BD (*p* = 0.002; *pc* = 0.02) (Fig. 2). For example, at the age of 20 years, 59% of those with both risk factors were already clinically affected as compared to the 37% of those without any risk factor. Co-occurrence of the *TLR2 rs*3804099 TT genotype (previously associated with early AAO) and reported low to severe sexual abuse had a statistically significant effect on determining early disorder-onset age when compared with any of the three other combinations.

## Discussion

It is well established that childhood traumatic experiences are associated with increased severity of BD, namely with an earlier age at onset (AAO), but possible interaction between environmental factors such as stress and genetic background is unknown [8]. Knowing that stress is a major inducer of inflammatory responses [11], we explored here the interaction between immunogenetic variations and presence of early and severe stress in a sample of BD patients. We observed a combined effect of both a genetic variant of *TLR2*, a major trigger of peripheral and central inflammatory responses, and reported childhood sexual abuse on the AAO, a proxy of increased severity of psychiatric and somatic manifestations in BD [7]. Thus, we propose that *TLR2 rs*3804099 TT genotype carriers may be more susceptible to inflammation-mediated damage induced by early stress with consequent earlier AAO of BD.

Early-life trauma is associated with permanent alterations of the immune system, namely development of chronic mild inflammation (CRP) [13] and stronger inflammatory responses (IL-6) subsequently to stress exposures [10]. Inflammation has also been suggested to be involved in the mechanisms underlying the comorbidity between metabolic syndrome and psychiatric disorders and causing premature mortality in individuals with a history of childhood adversity [11,43,44]. Childhood inflammation (IL-6) has been recently demonstrated to even precede the diagnosis of depression and psychosis in a prospectively followed general population cohort [45]. The genetic diversity of immune-related genes could thus be implicated in the inter-individual vulnerability to such stressors.

So far, genes linked to biological stress response systems *viz*. the hypothalamic-pituitary-adrenal axis (*FKBP5*), the monoaminergic system (*SLC6A4*, *COMT*) and the neurotrophic support of the CNS (*BDNF*), provided logical candidate genes to test for gene-environment interactions in several studies and confirmed the suspected genetic vulnerability and epigenetic changes to stressful/traumatic life events in psychiatric settings [46–53]. However, these molecules are not in direct relationship with the potential environmental risk factors as it is the case with TLR2, a sensor of BD-associated neurotropic pathogens [54–56]. This is of importance as prenatal immune priming through the TLR-mediated pathway and peripubertal stress exposure synergistically induced behavioral changes, unbalanced neurotransmitter levels and enhanced the expression of markers of inflammation and of microglia activation in stress sensitive brain areas of mice [35]. TLR2 molecules are pivotal both in homeostasis and in immune surveillance of the CNS [57]. Indeed, neuroinflammation can be elicited through the TLR2-mediated pathway either by endogenous molecules such as α-synuclein and amyloid β-peptide in Parkinson and Alzheimer disorders respectively [58,59] or by infectious insult as previously demonstrated for herpes simplex virus type 1 [60]. Moreover, associations between the *TLR2* genetic diversity and Alzheimer’s disease [61] or cognitive function in schizophrenia [62] are highly suggestive of genetically-driven inter-individual ability to modulate neuroinflammatory processes. Of importance is that the *TLR2 rs*3804099 polymorphism was showed on the one hand to be associated with several inflammatory and infectious diseases including pulmonary tuberculosis, neonatal infection, filariasis, ocular Behcet’s disease and cancer [63–67] and to exert a functional impact on the inflammatory response on the other [65,68,69].

It is plausible to hypothesize that *TLR2 rs*3804099 TT genotype carriers may be more susceptible to certain infectious insults and/or more prone to inflammation-mediated damage in presence of stress. TLR2, but not TLR4, has also been implicated in the perpetuation of inflammatory responses in the CNS after pro-inflammatory stimulation of astrocytes [70] and involved in hippocampal neurogenesis as mice lacking *TLR2* displayed impaired hippocampal and neuronal differentiation, which was not observed for *TLR4* [71]. These observations could be related to the *TLR2* specificity of our findings especially as hippocampal alterations have been repeatedly reported in adults with a history of childhood traumatic experiences and are suggested to be involved in the pathophysiology of BD [72–74].

Our regression analysis yielded a non-significant *TLR2*-CTQ interaction term, likely indicating that our results, while suggestive, are not robustly indicative of gene-environment interaction. Many genes with small effects are involved in the etiology of BD interacting with numerous environmental stressors. This may be one of the reasons for not detecting significance in regression analysis, leaving aside the limited sample size. However, a combined effect is observed in determining an earlier AAO in our patients when performing a Kaplan-Meyer survival curve. Since gender is a critical issue in BD as well as in the susceptibility to trauma, futures studies involving much more larger patient cohorts are warranted to address the issue. Indeed, we failed to observe any difference after sex-based stratification, possibly due to the sample size.

*TLR2* genetic susceptibility probably contributes to the neuro-immunological responses to pre- and perinatal infections establishing a lower threshold for subsequent stress-triggered pathological responses leading to a more severe clinical presentation. Such severity should be investigated in future studies including outcomes such as global functioning, cognition, suicidality, number and duration of mood episodes and development of comorbid conditions such as metabolic syndrome or autoimmune thyroiditis. Given the present findings, it would also be of interest to investigate the susceptibility conferred by this *TLR2* polymorphism to BD-associated pathogens like *Toxoplasma gondii* and the relationship between perinatal infections and childhood traumatic events on immune and clinical phenotypes of BD. Associations with infectious stigma, as well as markers of inflammation and stress components (e.g. cortisol) could not be explored as they were not available in our cohort. We deliberately chose to study euthymic patients to minimize the potential recall bias of traumatic childhood experiences associated with mood state. Moreover, the patients included in this study are from the mainland of France and of French descent with equal access to medical care (French social security system). Thus we cannot extrapolate these observations to BD in other populations socio-culturally and genetically distinct.

We propose that both risk factors, genetic polymorphism of *TLR2* and sexual abuse, independently found in earlier studies to be associated with earlier AAO, act cumulatively in immune-related pathways contributing to increase the “allostatic load” in BD. Further studies are warranted to sustain this model which may open the way to novel therapeutic targets and to personalized medicine based on one’s genetic background and history of exposure to environmental risk factors.
